# The impact of meteorological factors and PM2.5 on COVID-19 transmission

**DOI:** 10.1017/S0950268821002570

**Published:** 2022-01-21

**Authors:** Nan Zhou, HaoYun Dai, WenTing Zha, Yuan Lv

**Affiliations:** Key Laboratory of Molecular Epidemiology of Hunan Province, School of Medicine, Hunan Normal University, Changsha, Hunan, People's Republic of China

**Keywords:** Air pollution, COVID-19, SARS-CoV-2, temperature, wind speed

## Abstract

In this study, we analysed the relationship between meteorological factors and the number of patients with coronavirus disease 2019 (COVID-19). The study period was from 12 April 2020 to 13 October 2020, and daily meteorological data and the daily number of patients with COVID-19 in each state of the United States were collected. Based on the number of COVID-19 patients in each state of the United States, we selected four states (California, Florida, New York, Texas) for analysis. One-way analysis of variance ( ANOVA), scatter plot analysis, correlation analysis and distributed lag nonlinear model (DLNM) analysis were used to analyse the relationship between meteorological factors and the number of patients with COVID-19. We found that the significant influencing factors of the number of COVID-19 cases differed among the four states. Specifically, the number of COVID-19 confirmed cases in California and New York was negatively correlated with AWMD (*P* < 0.01) and positively correlated with AQI, PM2.5 and TAVG (*P* < 0.01) but not significantly correlated with other factors. Florida was significantly correlated with TAVG (positive) (*P* < 0.01) but not significantly correlated with other factors. The number of COVID-19 cases in Texas was only significantly negatively associated with AWND (*P* < 0.01). The influence of temperature and PM2.5 on the spread of COVID-19 is not obvious. This study shows that when the wind speed was 2 m/s, it had a significant positive correlation with COVID-19 cases. The impact of meteorological factors on COVID-19 may be very complicated. It is necessary to further explore the relationship between meteorological factors and COVID-19. By exploring the influence of meteorological factors on COVID-19, we can help people to establish a more accurate early warning system.

## Background

Coronavirus disease 2019 (COVID-19) was reported for the first time in Wuhan, China in December 2019 [1]. Many researchers have found that the COVID-19 is highly contagious [2]. On October 13, 2020, COVID-19 has caused a total of 7 781 174 confirmed infections and 214 426 deaths in the United States [3]. COVID-19 has spread to 210 countries and regions worldwide. The total number of COVID-19 cases and deaths is 37 704 153 and 1 079 029 (as reported by the WHO). Some studies have suggested that COVID-19 may be transmitted through aerosols containing the virus [4], indicating that the severe acute respiratory syndrome coronavirus 2 (SARS-CoV-2) can be transmitted by air.

The relationship between the number of COVID-19 patients and temperature has been studied in many regions, but the results differ. Studies in Singapore, Indonesia, Brazil, Norway and Japan found that temperature exhibited a positive correlation with COVID-19 transmission [5–9]. In the United States and Mexico, no significant relationships were noted [10, 11]; however, the number of COVID-19 patients and temperature were significantly negatively correlated in China, Iran, New York City and Bangladesh [12–15].

Many studies have shown that SARS-CoV-2 is more likely to spread through the air [16]. It has been suggested that the virus can reach long distances through atomisation. While a distance of 6 feet may not be achievable for SARS-CoV-2, studies have shown that microbes in droplets less than 5 μm in diameter may linger longer and can spread to people farther than 1 m [17]. The results of a study on Beijing air revealed that virus particles are presented in PM10 and PM2.5 in the air [18]. Studies have shown that activities lead to the formation of aerosols, which increase the chances of the spread of the SARS coronavirus [19]. A study of two hospitals in Wuhan found low concentrations of SARS-CoV-2 detected in aerosols in ventilated or isolation wards but high concentrations in closed rooms, such as toilets used by COVID-19 patients [20]. According to WHO data, approximately 4 million people die from outdoor air pollution every year. To reduce the number of deaths caused by air pollution every year, the WHO sets a guideline value for PM2.5. However, in many cities, this guideline value is often surpassed. One study found that seven deaths per 100 000 people in the United States and eight deaths per 100 000 people in France were linked to air pollution [21].

The global climate has changed dramatically since industrialisation and is likely to continue based on massive emissions of greenhouse gases, such as carbon dioxide, nitrogen dioxide, and other air pollutants, as well as the rapid development of agriculture. Meteorological factors have considerable effects on human health, and previous literature has shown the significant effects of meteorological factors on infectious diseases. There are doubts about whether the relationship between meteorological factors and the number of patients can describe the relationship between meteorological factors and disease transmission. However, many existing studies have used the number of patients for analysis and have obtained reliable results [10, 14, 22]. Therefore, we believe that in this study, the number of cases can be used as a reliable variable. In this study, we will explore the relationship between meteorological factors and COVID-19 in the United States and provide a reference for the COVID-19 epidemic.

## Methods

The meteorological data of the United States were collected from the National Centers for Environmental Information [23], including date, daily average wind speed (AWND), daily average precipitation (PRCP), daily average temperature (TAVG), daily PM2.5 (PM2.5) and daily AQI (AQI). The COVID-19 data of the United States were collected from the Coronavirus Resource Center of the JOHNS HOPKINS UNIVERSITY [3], which includes 7 781 174 of confirmed COVID-19 patients (Confirmed). The study period was from 12 April 2020 to 13 October 2020.

**Fig. 1.**
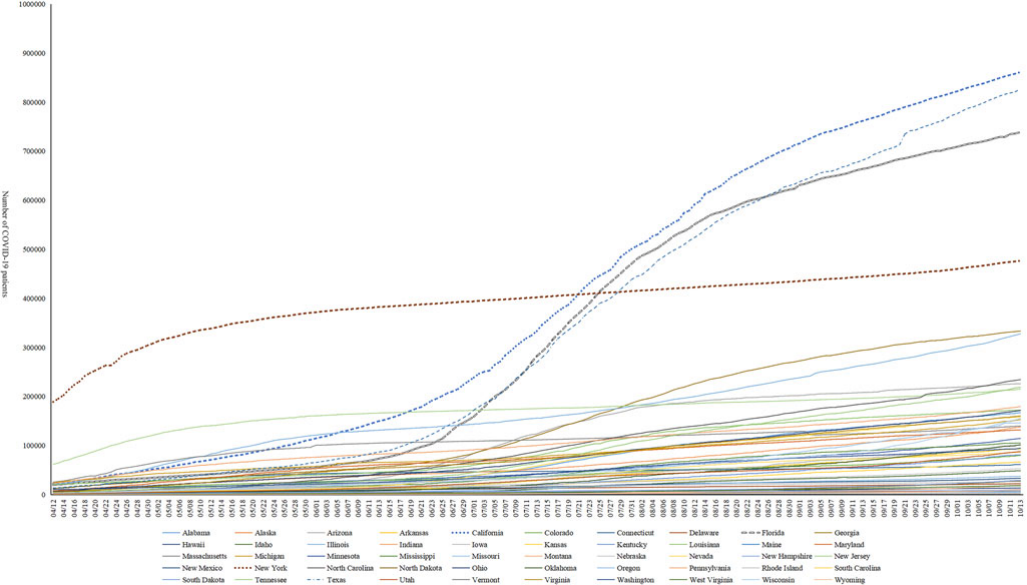
Changes in the number of COVID-19 patients in the United States.

We used Python 3 to clean the data (fill in the missing data, and remove variables with more missing data or variables unrelated to this study) and visually analyse the distribution of COVID-19 patients and meteorological factors in the United States. We conducted statistics on the COVID-19 epidemic in 50 states in the United States and selected the four states with the most COVID-19 patients from 12 April 2020 until 13 October 2020: California, Texas, Florida and New York (Fig. 1). The correlation between meteorological factors and the number of COVID-19 patients in the four states was analysed, and the Pearson correlation coefficient was calculated. According to the results of the correlation analysis, this research includes the three variables TAVG, PM2.5 and AWND and uses distributed lag nonlinear models (DLNMs) to analyse the relationship between TAVG, PM2.5, AWND and COVID-19 in the four states. First, we used DLNM to calculate the exposure-response relationship for each area. Then, we applied a multivariate meta-regression model to combine the overall effect estimates (in this study, Python 3 was used to preprocess the data, describe the variables and draw a scatter plot for each variable in the four regions studied). We constructed the DLNM model with R 4.0.5 and estimated the relevant parameters.

## Results

### The number of COVID-19 patients

California has the largest number of confirmed COVID-19 patients in the United States. There were 861 310 confirmed COVID-19 patients and 16 644 deaths due to COVID-19. According to the image, the number of confirmed COVID-19 patients in California, Texas and Florida increased until 15 June 2020 and slowed down around 12 August 2020, but an upward trend was still noted (Fig. 1). In New York, the number of confirmed COVID-19 patients did not increase significantly, but an accelerating upward trend was noted in October 2020 (Fig. 1).

### Meteorological Factors

**Fig. 2.**
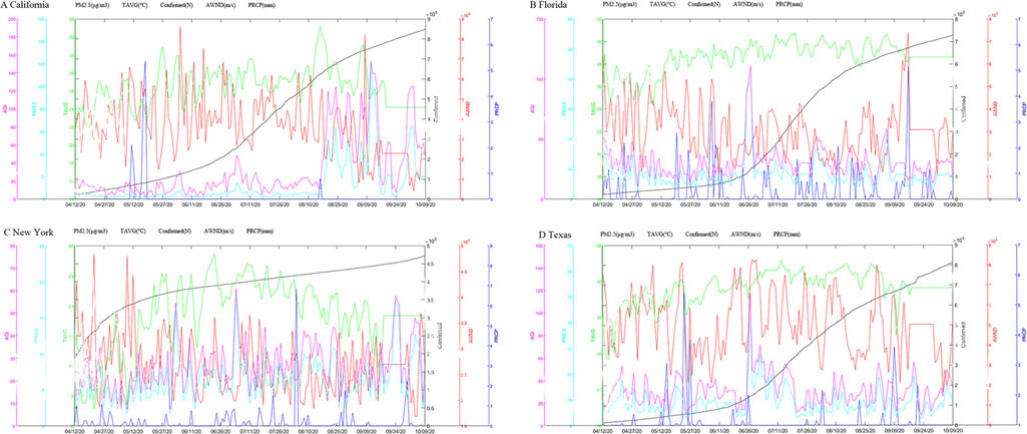
Changes in the number of COVID-19 patients and meteorological factors during the research in four states in the United States.

One-way ANOVA showed that all meteorological factors (AWND, PRCP, TAVG, PM2.5, AQI) were significantly different among the four states (*P* < 0.05) (Table 1). Pearson correlation analysis showed that the PRCP had no significant relationship with the number of COVID-19 patients in any state (*P* > 0.05) (Table 2). This result shows that rainfall does not directly affect the number of COVID-19 patients. However, rainfall may indirectly affect the number of COVID-19 patients by affecting the humidity of the air and affecting human activities. TAVG and AWND were significantly correlated with the number of COVID-19 patients in most situations (*P* < 0.05) (Table 2), suggesting that TAVG and AWND had a greater influence on the number of COVID-19 patients than PRCP. Temperature can affect the survival of the virus, and wind speed can affect the spread of the virus. However, the effect of rain on the survival and spread of the virus and subsequently the number of COVID-19 patients is unclear. Moreover, from 12 April 2020 to 13 October 2020, the average rainfall in the four states ranged from (0.05 ± 0.32 mm) to (6.00 ± 13.18 mm) (Fig. 2). PM2.5 and AQI were significantly correlated with the number of COVID-19 patients in two states (*P* < 0.05) (Table 2). Specifically, in California and New York, PM2.5 and AQI began to increase significantly after August, and the temperature and wind speed in New York and California also began to decrease after August. This result suggests that PM2.5 and AQI may have an impact on the spread of COVID-19, but this effect is related to the local environment and human activities.

**Table 1.** Difference in meteorological factor between four states

Variable	States				Statistical Prarameter	
	California	Florida	New York	Texas	*F*	*P*-Value
AWND(m/s)	2.83 ± 1.07	2.60 ± 0.98	3.21 ± 1.50	3.94 ± 1.24	42.62	<0.001
PRCP(mm)	0.05 ± 0.32	6.00 ± 13.18	2.60 ± 8.66	1.52 ± 5.70	16.88	<0.001
TAVG(°C)	22.58 ± 3.62	25.41 ± 2.76	19.26 ± 6.08	26.99 ± 3.74	118.94	<0.001
PM2.5(μg/m^3^)	14.56 ± 22.77	10.24 ± 6.18	8.01 ± 3.61	10.47 ± 6.35	9.06	<0.001
AQI	42.02 ± 44.99	39.83 ± 17.32	32.61 ± 13.02	40.32 ± 18.17	4.54	<0.05

**Table 2.** Pearson correlation between meteorological factors and number of COVID-19 patients in four states

STATION	Variable	Confirmed (N)	AWND (m/s)	PRCP (mm)	TAVG (°C)	PM2.5 (μg/m^3^)	AQI
California	Confirmed	1	−0.275**	−0.140	0.440**	0.546**	0.647**
	AWND	−0.275**	1	0.168*	−0.237**	−0.414**	−0.425**
	PRCP	−0.140	0.168*	1	−0.167*	−0.081	−0.110
	TAVG	0.440**	−0.237**	−0.167*	1	0.154*	0.243**
	PM2.5	0.546**	−0.414**	−0.081	0.154*	1	0.950**
	AQI	0.647**	−0.425**	−0.110	0.243**	0.950**	1
Florida	Confirmed	1	−0.089	0.019	0.278**	−0.054	−0.063
	AWND	−0.089	1	0.225**	−0.286**	−0.198**	−0.226**
	PRCP	0.019	0.225**	1	−0.068	−0.178*	−0.221**
	TAVG	0.278**	−0.286**	−0.068	1	0.189*	0.176*
	PM2.5	−0.054	−0.198**	−0.178*	0.189*	1	0.979**
	AQI	−0.063	−0.226**	−0.221**	0.176*	0.979**	1
New York	Confirmed	1	−0.379**	0.024	0.674**	0.316**	0.325**
	AWND	−0.379**	1	0.080	−0.274**	−0.403**	−0.435**
	PRCP	0.024	0.080	1	0.041	−0.118	−0.120
	TAVG	0.674**	−0.274**	0.041	1	0.388**	0.412**
	PM2.5	0.316**	−0.403**	−0.118	0.388**	1	0.986**
	AQI	0.325**	−0.435**	−0.120	0.412**	0.986**	1
Texas	Confirmed	1	−0.302**	−0.061	0.111	−0.077	−0.062
	AWND	−0.302**	1	0.093	0.264**	0.275**	0.241**
	PRCP	−0.061	0.093	1	−0.075	−0.081	−0.098
	TAVG	0.111	0.264**	−0.075	1	0.174*	0.180*
	PM2.5	−0.077	0.275**	−0.081	0.174*	1	0.976**
	AQI	−0.062	0.241**	−0.098	0.180*	0.976**	1

*P < 0.05;**P < 0.01.

The significant influencing factors of the number of COVID-19 cases differed among the four states. Specifically, the number of COVID-19 confirmed cases in California and New York was negatively correlated with AWMD (*P* < 0.01) and positively correlated with AQI, PM2.5, and TAVG (*P* < 0.01) but not significantly correlated with other factors. Florida was significantly affected by TAVG (positive) (*P* < 0.01) but not significantly affected by other factors. The number of COVID-19 cases in Texas was only significantly negatively associated with AWND (*P* < 0.01) (Table 2). The study analysed four different regions in the United States, where environmental factors such as temperature, light, wind speed and humidity, may have affected the spread of the virus. Moreover, seasons and climate also affect human activities, such as travel and gatherings. Low temperatures may restrict human travel, and comfortable temperatures may encourage humans to hold gatherings.

### Distributed Lag nonlinear models (DLNM)

**Fig. 3.**
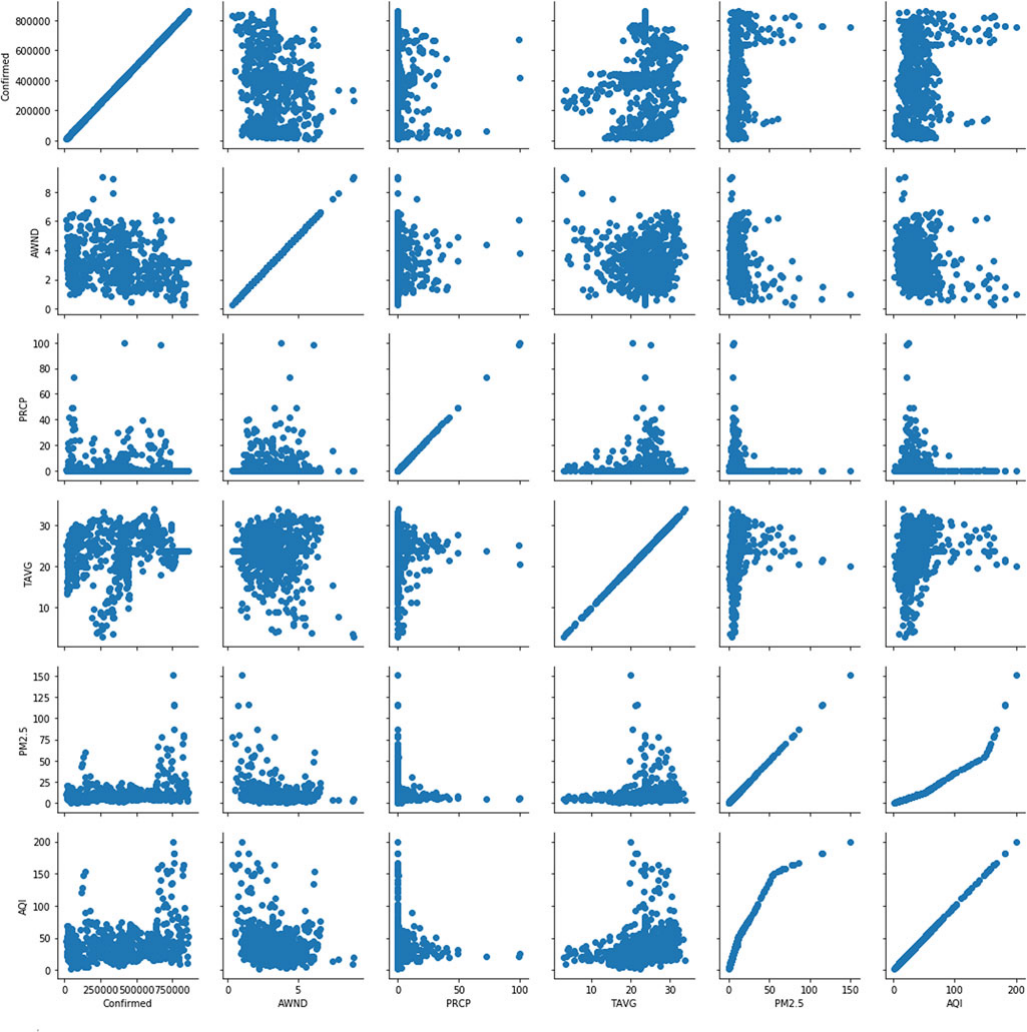
Scatter plot of the number of confirmed COVID-19 patients and meteorological factors in four regions (California, Florida, New York, and Texas).

Here, *α* is the intercept; *β* is the regression coefficient; 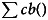 represents the two-dimensional matrix of meteorological factors and lag days. The natural cubic spline function with 3 degrees of freedom was used; we defined 14 days as the maximum lag days. In addition, *ns()* denotes the smoother based on natural regression splines; TAVG, PM2.5, AWND are the three-day moving TAVG (*df* = 6), PM2.5 (*df* = 3), and AWND(*df* = 3), respectively. Here, *time* refers to the long-term trend of the time, and *dow* indicates the day of the week, which was controlled as a categorical variable. Here, *t* is the observation date; *j* refers to the regions; and *E(Y_tj_)* is the expected value of the number of COVID-19 cases in region *j* on Day *t*. The PRCP is not significant in all states. PM2.5 and AQI are highly correlated (Fig. 3), so PRCP and AQI are excluded. The modified DLNM models are shown in equations (1–3).1
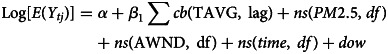
2
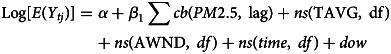
3
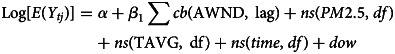

After establishing the DLNM model, we examined the cumulative lag effects of meteorological factors at TAVG, PM2.5 and AWND on the number of COVID-19 cases under lag exposure (lag_TAVG_ and lag_PM2.5_ is 04, lag_AWND_ is 07) [24].

**Fig. 4.**
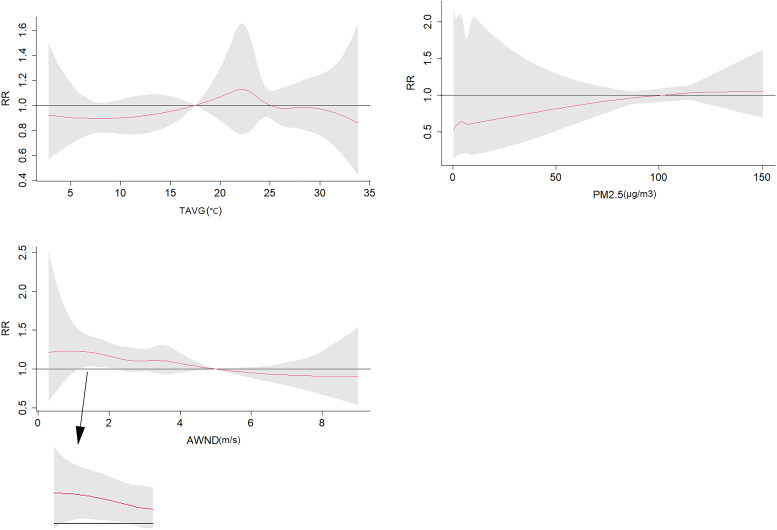
Exposure-response curve (TAVG, PM2.5, and AWND).

The results of the model found that temperature and PM2.5 had no effect on the number of COVID-19 cases. When the wind speed is approximately 2 m/s, the wind speed has a positive relationship with the spread of COVID-19 (Fig. 4).

## Discussion

We collected the number of COVID-19 patients and meteorological data in various states in the United States from 12 April 2020 to 13 October 2020 and used one-way ANOVA, scatter plots, Pearson correlation coefficients, and DLNM methods to analyse COVID-19. Data from the four states with the largest number of patients were analysed. One-way ANOVA reveals significant differences in the meteorological data of the four states. During the period from 12 April 2020 to 13 October 2020, the four states received less rainfall, and the average temperature was less than 30 °C. New York had the lowest AQI, and California had the highest AQI. We found that temperature was positively correlated with the number of COVID-19 patients in many regions. In addition, the wind speed was negatively correlated with the number of COVID-19 patients in many regions, and PM2.5 and AQI were positively correlated with the number of COVID-19 patients in most regions. During the construction of the DLNM, because the Pearson correlation coefficient and the scatter plot showed that the rainfall in the four regions did not show a correlation with the number of COVID-19 patients and a strong correlation was noted between PM2.5 and the AQI, the model incorporated temperature (TAVG), wind speed (AWND) and PM2.5. The model results show that after combining the DLNM results of the four regions with a meta-analysis random-effects model, the impact of temperature and PM2.5 on COVID-19 is not obvious. When the wind speed was 2 m/s, a significant positive correlation with COVID-19 cases was noted.

According to research on virus transmission, the occurrence of viral infections is generally linked to the concentration of air pollution particles [25]. In most cases, atmospheric particulate matter can serve as a carrier of most viruses or bacteria in the air. Another study described the possible relationship between the spread of COVID-19 in Italy and exceeding the PM10 limit [26]. Related experimental results show that droplet particles and atomised particles in the air have a long diffusion time and diffusion distance in air, so it can be considered that the fine particles in the air can be virus carriers. From an epidemiological point of view, a comparison of 107 provincial and 20 regions in Italy finally found an increase in the hospitalisation rate with a higher average concentration of PM2.5 [27], and the same result was noted in the Wuhan study. After the outbreak, both places were strictly sealed off. The lockdown not only isolated COVID-19 cases from unaffected people but also reduced air pollution and reduced PM2.5 levels in Wuhan by 44% [28]. In contrast, Sweden did not implement a blockade, and its PM2.5 content is also quite low. Since 2020, the PM2.5 content in Sweden is generally less than 25 μg/m^3^. In addition, there were only 22 721 COVID-19 cases and 2769 deaths [29]. However, there is no clear dose–response relationship, and it is impossible to meet all the causality standards, so it is impossible to determine the effect of the concentration of airborne particles.

Based on the analysis of climate variables, evidence suggests that temperature affects influenza epidemics in tropical regions [30]. The temperate regions of the Northern and Southern Hemispheres experience highly synchronised annual influenza epidemics every month in winter [31]. The seasonality of influenza in temperate monsoon climate regions may be caused by meteorological factors that affect the environmental and physical stability of virus particles and human social behaviour, both of which contribute to the dynamics of virus epidemiology. At present, the peak incidence of most respiratory diseases is accompanied by seasonal fluctuations. Related studies have shown that pathogens will be wrapped in respiratory mucus after entering the human respiratory tract and eliminated by the cilia of epithelial cells. This process is called mucociliary clearance (MCC), and dry cold air significantly affects this protection mechanism [32]. Although this study found that a specific temperature increase would lead to an increase in the number of COVID-19 patients, during the study period, the highest average temperature in these four areas was only approximately 32 °C. Therefore, an increase in temperature within a certain range may lead to increased molecular movement in the air, thereby increasing the number of COVID-19 patients.

COVID-19 is mainly spread by the air [4]. In previous studies, it was easy to overlook the impact of wind speed on the spread of disease. We found that low wind speed(2 m/s) had a significant positive correlation with the spread of COVID-19, which may be related to the wind speed affecting the spread of the virus.

## Conclusions

We used one-way ANOVA, scatter plots, Pearson correlation coefficients, and DLNM to analyse the relationship between the number of COVID-19 patients and meteorological conditions in the United States. Temperature, wind speed, PM2.5 and AQI all showed a significant correlation with the number of COVID-19 patients, but the influence of temperature and PM2.5 on the spread of COVID-19 was not obvious. This study shows that when the wind speed was 2 m/s, it had a significant positive correlation with COVID-19 cases. It is necessary to further analyse and explore the relationship between meteorological factors and COVID-19. By exploring the influence of meteorological factors on COVID-19, we can help people to establish a more accurate early warning system.

## Data Availability

Data sources for this study: https://ourworldindata.org/, https://coronavirus.jhu.edu/data.
