# The impact of meteorological factors and PM2.5 on COVID-19 transmission – CORRIGENDUM

**DOI:** 10.1017/S0950268822001406

**Published:** 2022-09-14

**Authors:** Nan Zhou, HaoYun Dai, WenTing Zha, Yuan Lv

**Affiliations:** Key Laboratory of Molecular Epidemiology of Hunan Province, School of Medicine, Hunan Normal University, Changsha, Hunan, People's Republic of China

A funding acknowledgement was missing from the original publication of this article. The below acknowledgement should have been included

Fund Name: Key Project of Hunan Provincial Science and technology innovation [No. 2020SK1015-3]

This has now been added to the original version of record.
